# Two Predicted Transmembrane Domains Exclude Very Long Chain Fatty acyl-CoAs from the Active Site of Mouse Wax Synthase

**DOI:** 10.1371/journal.pone.0145797

**Published:** 2015-12-29

**Authors:** Steffen Kawelke, Ivo Feussner

**Affiliations:** 1 Department for Plant Biochemistry, Albrecht-von-Haller-Institute for Plant Sciences, Georg-August-University, Justus-von-Liebig-Weg 11, 37077 Goettingen, Germany; 2 Department for Plant Biochemistry, Goettingen Center for Molecular Biosciences (GZMB), Georg-August-University, Justus-von-Liebig-Weg 11, 37077 Goettingen, Germany; 3 Department for Plant Biochemistry, International Center for Advanced Studies of Energy Conversion (ICASEC), Georg-August-University, Justus-von-Liebig-Weg 11, 37077 Goettingen, Germany; USDA-ARS, UNITED STATES

## Abstract

Wax esters are used as coatings or storage lipids in all kingdoms of life. They are synthesized from a fatty alcohol and an acyl-CoA by wax synthases. In order to get insights into the structure-function relationships of a wax synthase from *Mus musculus*, a domain swap experiment between the mouse acyl-CoA:wax alcohol acyltransferase (AWAT2) and the homologous mouse acyl-CoA:diacylglycerol O-acyltransferase 2 (DGAT2) was performed. This showed that the substrate specificity of AWAT2 is partially determined by two predicted transmembrane domains near the amino terminus of AWAT2. Upon exchange of the two domains for the respective part of DGAT2, the resulting chimeric enzyme was capable of incorporating up to 20% of very long acyl chains in the wax esters upon expression in *S*. *cerevisiae* strain H1246. The amount of very long acyl chains in wax esters synthesized by wild type AWAT2 was negligible. The effect was narrowed down to a single amino acid position within one of the predicted membrane domains, the AWAT2 N36R variant. Taken together, we provide first evidence that two predicted transmembrane domains in AWAT2 are involved in determining its acyl chain length specificity.

## Introduction

**Fig 1:**
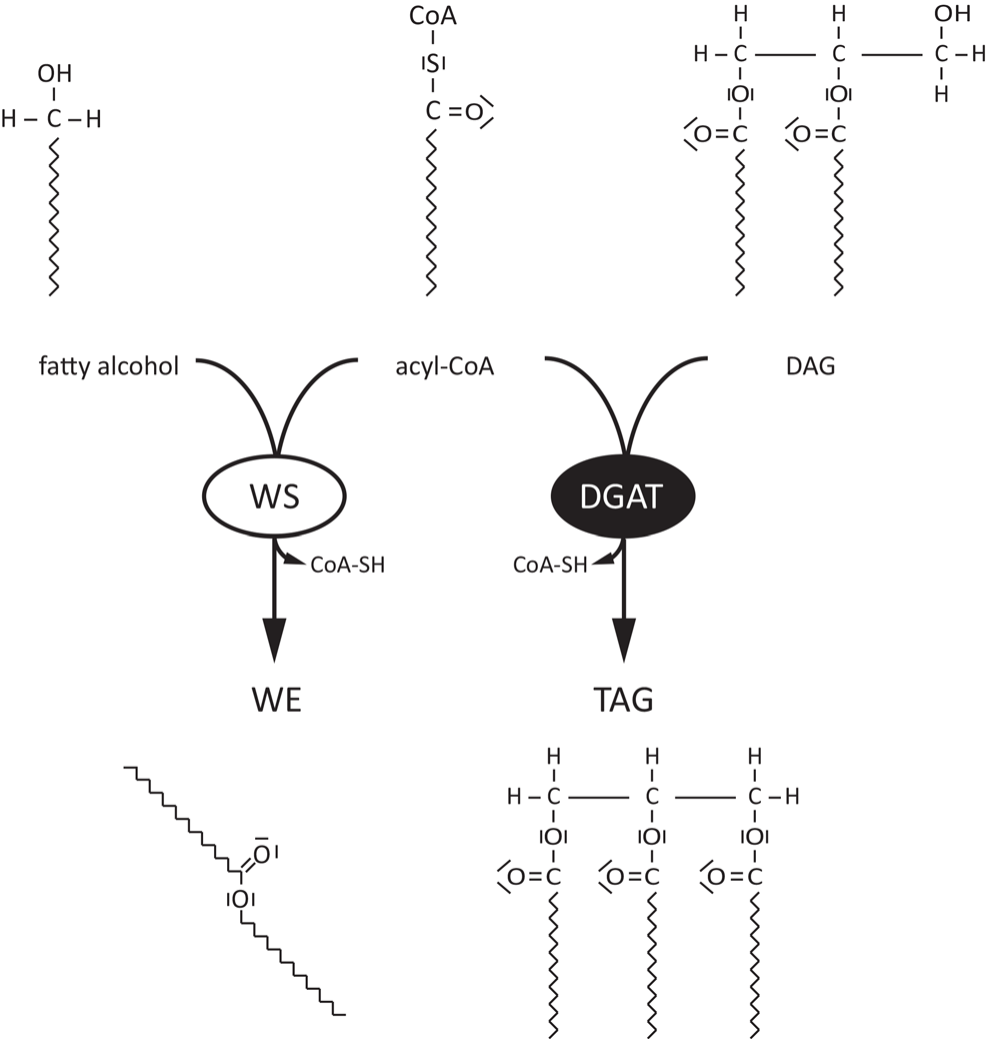
Comparison of WS and DGAT enzyme reaction. Wax synthases (WSs) or acyl-CoA:wax alcohol acyltransferases catalyze the condensation of a fatty alcohol with an acyl-CoA, thereby forming wax esters (WEs). In contrast, acyl-CoA:diacylglycerol O-acyltransferases (DGATs) catalyze the condensation of an acyl-CoA molecule with diacylglycerol (DAG) to yield triacylglycerols (TAGs).

Wax esters (WEs) are used as coatings or beside triacylglycerols (TAGs) as storage lipids in all kingdoms of life [1]. While plants primarily use WEs for sealing their surfaces and bacteria use them for energy storage, in mammals WEs are important constituents of the meibomian and sebaceous gland lipids that play a critical role in protecting the human eye and skin against desiccation [2,3]. Two enzymes are needed for WE synthesis: a fatty acyl reductase (FAR) and a wax synthase (WS). The first enzyme, FAR, provides the acyl acceptor moiety of a WE through formation of a fatty alcohol via a NAD(P)H-dependent reduction of a fatty acyl-CoA molecule [4]. This reaction releases a first CoA molecule. The alcohol is then esterified to a second acyl-CoA molecule by the action of a WS, yielding the WE molecule and a second molecule of CoA (Fig 1) [5].

According to their sequence homologies, WSs can be divided into three distinct subclades. The first group includes type 1 and type 2 mammalian acyl-CoA:wax alcohol acyltransferases (AWATs) [6], the second Jojoba WS-like sequences [1] and the third bacterial WSs of the bifunctional wax synthase/acyl-CoA:diacylglycerol O-acyltransferases (DGAT)-type (WSD-type) [7]. None of these subclades share considerable sequence homology with each other. Most WSs described so far exhibit a rather broad substrate spectrum, which consequently results in a heterogeneous mixture of synthesized WEs [8,9,10]. In addition, knowledge about structure-function relationships within all known WSs is scarce and to date there is only one study which describes the modification of the substrate specificity of a WS. A change in the preference of a WSD from *M*. *aquaeolei* (MaWSD1) and *A*. *baylyi* (AbWSD1) for fatty alcohols was achieved by exchanging a single amino acid in both enzymes. The crucial residue was identified through alignments of crystal structures of the polyketide-associated protein A5 (PapA5) and several substrate-bound carnitine acetyltransferases. In a second step, the corresponding residues in the WSD was identified by sequence alignment of AbWSD1 and PapA5, respectively [11]. By contrast, no data about structure function-relationships in respect to substrate specificity are available for WSs of the AWAT1/AWAT2 or Jojoba-type. In case of AWAT2-type WSs, the best studied enzyme is the one from *Mus musculus* [12], whose substrate specificity was already described in detail [8]. When expressed in yeast, it showed a broad substrate specificity having the highest activity for 12:0-CoA, 14:0-CoA, and 16:0-CoA in the presence of medium chain alcohols. Unsaturated alcohols longer than C18 were better utilized than saturated ones. However, when the enzyme was expressed in Arabidopsis seeds it showed a preference towards C18 and C20 monounsaturated fatty alcohols and polyunsaturated C18 acyl-CoA substrates [13,14]. In the present study, the substrate specificity of this enzyme was analyzed by a domain swap experiment. Two predicted transmembrane (TM) domains of mouse AWAT2 turned out to be involved in determining the enzyme’s substrate and product specificity.

## Experimental procedures

### Materials

Restriction enzymes and DNA-modifying enzymes were obtained from MBI Fermentas. Standards of Fatty acyl-CoAs, fatty alcohols and wax esters were obtained from Sigma, Nu-Chek-Prep, or Avanti. Methanol, chloroform, n-hexane, and iso-propanol (all HPLC grade) as well as all other chemicals were from Sigma. Basic molecular biological and biochemical techniques were performed as described [15].

### Cloning of mouse AWAT2 and mouse DGAT2 in pYES2/NT

Primers used for cloning of the DNA constructs used in this study are depicted in S1 Fig. For cloning of sequences into the expression vector pYES2/NT (Invitrogen), BamHI and XhoI recognition sites were added to the forward and reverse primers, respectively. In case of the AWAT2 coding sequence, a codon optimized version for *Brassicaceae* was used as described previously [14]. The coding sequence of mouse acyl-CoA:diacylglycerol O-acyltransferase 2 (DGAT2) was amplified from liver-derived mouse cDNA using the Phusion DNA polymerase (Thermo Fisher Scientific) and a standard PCR protocol: 98°C for 4 min, followed by 18 cycles at 57°C for 60 sec, 72°C for 30 sec/kb, 98°C for 60 sec and a terminal elongation step at 72°C for 10 min. All sequences were verified by DNA sequencing.

### Construction of domain swap variants

Domain swap variants were constructed via overlap extension PCR after amplification of respective fragments from wild type sequences of AWAT2 and DGAT2. In case of AWAT2, fragments were chosen according to the predicted topology of the enzyme. Respective data was generated using the services of TMHMM [16], SOSUI [17] and Phobius [18]. In case of DGAT2, the membrane topology was experimentally elucidated before [19]. Thus, fragments were chosen according to the results of Stone et al. [19]. All primers for the overlap extension PCRs were designed with a 20 bp overhang corresponding to the adjacent fragment in case of forward primers and a 15 bp overhang in case of reverse primers. Amplified fragments were purified via gel extraction and subsequently used for overlap extension PCR using a standard PCR protocol (see above).

### Construction of single amino acid exchange variants

All single amino acid exchange variants were created by site directed mutagenesis [20]. Primers were designed as follows: ~10 bases in direction of 5’-end of the mutation, ~20 bases in direction of 3’-end of the mutation, ~10 non-overlapping bases at the 3‘-end of both primers. In all cases, pYES2/NT constructs of AWAT2 and DGAT2 were used as a template.

### Cultivation of *S*. *cerevisiae* H1246

Transformation of the expression constructs in yeast was done as described previously [21]. For cultivation, 20 ml of single dropout media lacking uracil and supplemented with 2% of galactose were inoculated with a respective overnight culture to a final OD_600_ = 0.05. Fatty alcohols were fed to a final concentration of 1 mM. Expression cultures were incubated for five days at 30°C while shaking at 180 rpm.

### Lipid extraction

Fifty OD_600_-units of yeast cells were harvested by centrifugation, resuspended in 1 ml methanol and vortexed for 15 min together with 0.5 mm glass beads. After addition of 2 ml *n-*hexane, samples were vortexed for another 15 minutes. Polar and organic phase were separated by centrifugation at 500 x g for 10 minutes. The organic phase was transferred to a new tube and evaporated under a stream of nitrogen. Samples were then dissolved in 200 μl of *n-*hexane and transferred into GC vials for further analyzes.

### Thin layer chromatography

For thin layer chromatography (TLC) analyzes, neutral lipid extracts from yeast were spotted on a TLC plate using an automatic TLC sampler (Camag). The TLC was developed with *n-*hexane:diethyl ether:acetic acid (80:20:1, *v/v/v*). Separated lipids were visualized by soaking the plates with CuSO_4_ and subsequent heating to 190°C. The WE/TAG ratios, meaning the ratios of the intensities of the spots for WEs and TAGs were determined densitometrically using the ImageJ software [22].

### GC-MS analyzes

Initial identification of single WE species was performed by GC-MS. Lipid extracts from yeast were dissolved in 200 μl *n-*hexane and 2 μl of this sample were subjected to GC-MS analysis using a Polaris Q mass selective detector connected to a Trace gas chromatograph (Thermo Finnigan) equipped with a Restek Rxi™-5ms capillary column (15 m x 0.25 mm, 0.25 μm film thickness; Restek). Helium was used as the carrier gas (1.5 ml min^-1^). The temperature gradient was 2 min at 60°C, 60-200°C at 40°C min^-1^, 2 min at 200°C, 200–340°C at 3°C min^-1^ and 340°C for 16 min. The WEs were detected by electron impact ionization (-70 eV, ion source 200°C, Aux-line 350°C) in a mass range of 50–730 amu.

### GC-FID analyzes

For relative quantification of WE species, yeast WE preparations were dissolved in 200 μl *n-*hexane and 2 μl of this sample were subjected to GC-FID using a 6890 Series GC System (Agilent) equipped with an Agilent 19091j-413 HP5 5% Phenyl Methyl Siloxane column (30 m x 320 μm x 0.25 μm film thickness; Agilent). Helium was used as the carrier gas (1.5 ml min^-1^). The split ratio was 5:1. The temperature gradient was 2 min at 60°C, 60–200°C at 40°C min^-1^, 2 min at 200°C, 200–325°C at 3°C min^-1^ and 325°C for 16 min. WEs were detected by flame ionization detection.

### Acyl-CoA extraction and analysis

Acyl-CoA extraction was done according to [23]. Briefly, yeast cells corresponding to 80 OD_600_ units were extracted by vortexing with 3 ml chloroform methanol mixture (2:1, *v/v*) and glass beads. After addition of 1 ml chloroform and 1 ml water, the aqueous and organic phases were discarded, whereas the interphase was dried under a stream of nitrogen. After addition of 400 μl extraction buffer (2 ml isopropanol, 2 ml KH_2_PO_4_, pH 7.2, 50 μl acetic acid, 80 μl BSA (50 mg/ml in H_2_O, fatty acid free)), 10 μl saturated (NH_4_)_2_SO_4_ and 1.2 ml methanol chloroform mixture (2:1, *v/v*), samples were briefly vortexed and incubated for 20 min at room temperature. After centrifugation to remove debris, the supernatant was dried under nitrogen and derivatized with 200 μl derivatization buffer (0.5 M chloroacetaldehyde, 0.15 M citrate buffer; pH 4.0, 0.5% SDS (*w/v*)) at 85°C for 20 min. Samples were then cooled down to 4°C for 30 min and further analyzed by HPLC [24]. The acyl-CoA species were assigned to their molecular identity by coelution with known standard substances. In case of 24:1-CoA and 26:1-CoA, no standards were available and the molecular nature was tentatively assigned in analogy to the elution pattern of 16:1-CoA—22:1-CoA. Relative quantification of single acyl-CoA species was done according to [24].

## Results

### Mouse AWAT2 and mouse DGAT2 are predicted to share a similar topology

**Fig 2:**
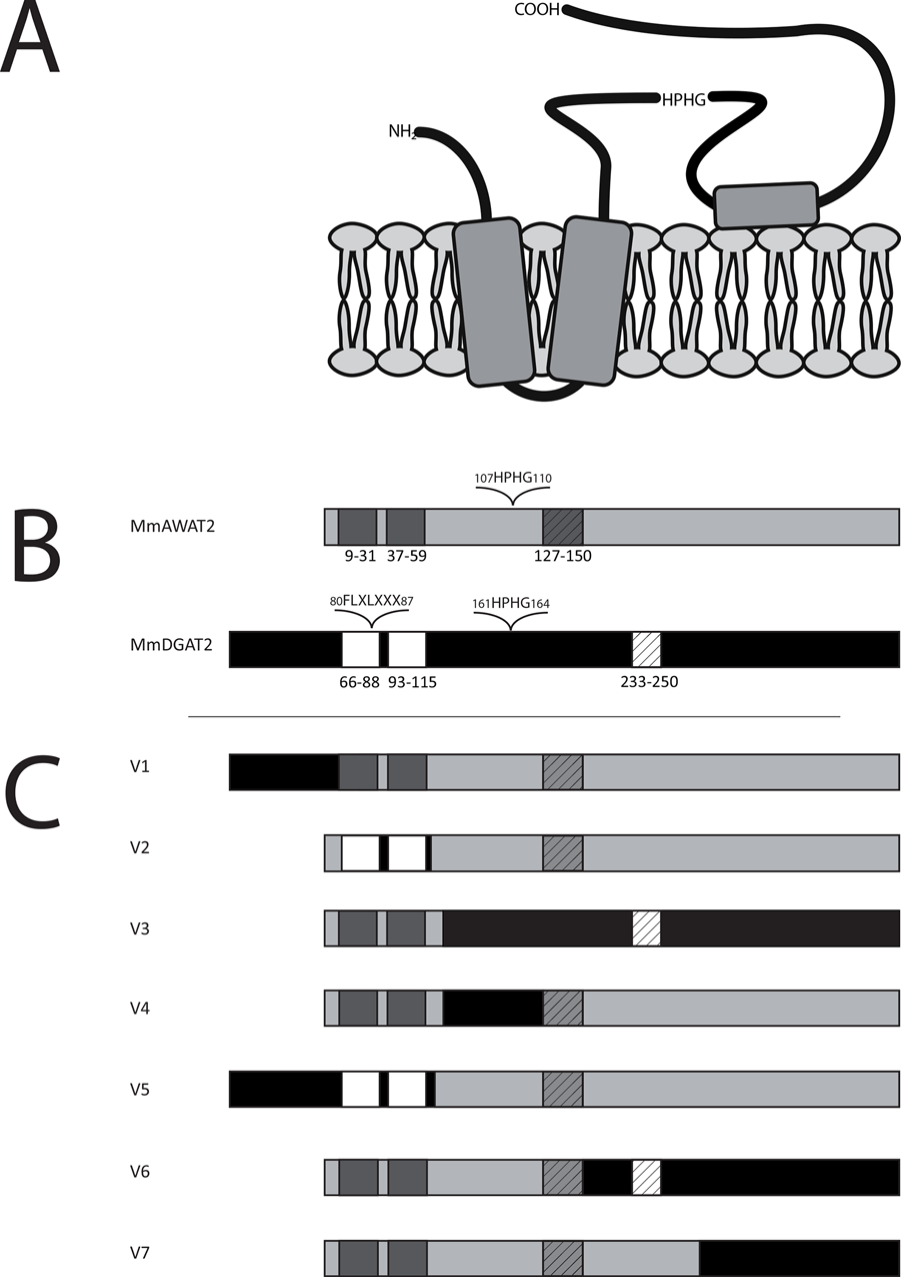
A. Topological model of AWAT2 in accordance to data generated by the services of Phobius [18], TMHMM [16] and SOSUI [17]. According to the model, AWAT2 is predicted to contain an N-terminal cytoplasmic tail that is linked to two TM domains, which are connected by a short linker. The remaining C-terminal sequence forms again a cytosolic domain, which contains the catalytic HPHG motif of the enzyme. A hydrophobic patch that may form a membrane contact is located in the middle of this domain. B. Predicted domain structures of mouse AWAT2 and mouse DGAT2. The conserved TM domains (dark grey/white boxes), the hydrophobic patch (hatched boxes), the active site motif HPHG and the putative neutral lipid binding motif “FLXLXXX” of DGAT2 are indicated. C. Domain swap variants of mouse AWAT2 and mouse DGAT2. Again, the conserved TM domains (dark grey/white boxes) and the hydrophobic patch (hatched boxes) are indicated. The sequences of mouse AWAT2 and mouse DGAT2 were used to construct seven domain swap variants (V1-V7), in which different parts of mouse AWAT2 were exchanged for the respective parts of mouse DGAT2. Mouse DGAT2 derived parts are shown in black (non-TM domains) and in white (TM-domains), respectively.

Mouse AWAT2 (hereafter referred to as “AWAT2”) and mouse DGAT2 (hereafter referred to as “DGAT2”) are both acyltransferases of the DGAT2-type, which share an overall sequence similarity of 76%. Despite these relatively high similarities on the sequence level, the main products formed by the two enzymes are reported to be different [8,19]. The aim of this study was to evaluate the underlying molecular basics of the differential acyltransferase-activity by a domain swap approach and directed mutagenesis. The domain structure of DGAT2 was analyzed in detail previously [19]. It consists of a cytoplasmatic tail, which is linked to two N-terminal transmembrane helices, of which the first one harbors a FLXLXXX motif, which may be involved in neutral lipid binding (Fig 2A and 2B) [25,26]. The two helices are connected to each other by a short linker. The C-terminal part of DGAT2, which also contains the catalytic HPHG motive, is localized in the cytoplasm (Fig 2B). In order to analyze if the similarity of DGAT2 and AWAT2 on their sequence level is reflected by their domain structure, three different programs (Phobius [18], TMHMM [16] and SOSUI [17]) were used for the prediction of the domain structure of AWAT2. Overall, all programs revealed a topology model similar to that of DGAT2. As DGAT2, AWAT2 is predicted to contain an N-terminal cytoplasmic tail that is linked to two TM domains, which are connected by a short linker (Fig 2A). The remaining C-terminal sequence forms again a cytosolic domain, which contains the catalytic HPHG motif of the enzyme. A hydrophobic patch that may form a membrane contact is located in the middle of this domain [19] (Fig 2A).

### Three domain swap variants of AWAT2 and DGAT2 yielded active WS variants

In order to identify determinants for the substrate and product specificity of AWAT2, chimeric variants were generated by exchanging different segments between AWAT2 and DGAT2. For this, both sequences were divided into five domains on the basis of their membrane topology according to the following pattern: The N-terminal cytosolic tail, the two TM domains, the catalytic domain including the hydrophobic patch and the remaining C-terminal part, divided into two parts. By exchanging the different segments, seven domain swap variants were constructed and named V1-V7 (Fig 2C).

In the course of this study, AWAT2, DGAT2 as well as V1—V7 were expressed in a quadruple knockout mutant strain *S*. *cerevisiae* H1246, which is devoid of all four genes responsible for neutral lipid synthesis [27]. Thus, all neutral lipids detected in this strain upon expression of heterologous genes can directly be assigned to the corresponding gene products. Since *S*. *cerevisiae* does not possess an intrinsic FAR activity [28], fatty alcohol was fed to the cultures in order to enable wax ester synthesis. Consequently, all synthesized WEs carried the supplemented fatty alcohol moiety, whereas the acyl chain distribution of the respective WE profiles was determined by the substrate specificity of the WE synthesizing enzyme and the endogenous acyl-CoA pool. All constructs were expressed in yeast cultures supplemented with either 16:0-OH or 18:1-OH. The corresponding WE fractions were analyzed by TLC.

**Fig 3:**
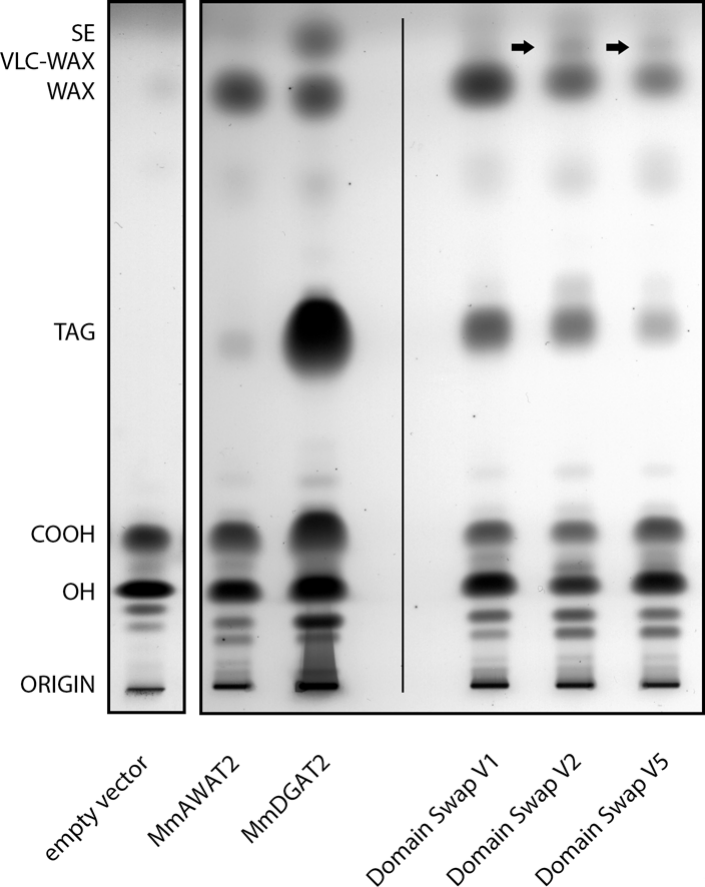
Separation of neutral lipids derived from yeast cultures expressing different mouse AWAT2 variants, an empty vector control, AWAT2 wt or DGAT2 wt. pYES2/NT empty vector control (left side), mouse AWAT2 and DGAT2 wild type enzymes (middle) as well as domain swap variants derived from those two enzymes (right) were expressed in *S*. *cerevisiae* H1246 and the cultures were supplemented with 18:1-OH. The left part of the figure, which shows the empty vector control, originates from a different TLC plate than the rest of the shown samples do. Similar results were obtained when cultures were supplemented with 16:0-OH (not shown). VLC fatty acyl-containing WEs are indicated by arrows. COOH = free fatty acids, OH = fatty alcohols, TAG = triacylglycerols, WE = wax esters, VLC WE = very long chain fatty acyl-containing wax esters, SE = sterol esters. Data are representative for at least three independent biological replicates.

As expected, *S*. *cerevisiae* H1246 cultures expressing an empty vector control accumulated no WEs in the presence of fatty alcohols (Fig 3, left lane). AWAT2 predominantly formed WEs as well as minor amounts of TAG upon expression in the presence of fatty alcohols (Fig 3). DGAT2 on the other hand predominantly formed TAGs, but was also capable to synthesize WEs and substances migrating like sterol esters when fatty alcohols were present (Fig 3). However, in comparison to the amount of TAG, the amount of wax synthesized by the respective cultures was relatively low. While we confirmed the presence of WEs by GC/MS, the identity of the band migrating like a sterol ester standard was not further analyzed by any other method.

In case of the domain swap variants V1—V7, only expression of V1, V2 and V5 resulted in substantial neutral lipid synthesis. Variants V3, V4, V6 and V7 did not mediate WE or TAG formation upon expression in *S*. *cerevisiae* (not shown). All active variants contained the complete C-terminal part of AWAT2 beginning behind the two predicted TM domains, whereas in all inactive variants, this part was altered (Fig 2C).

**Fig 4:**
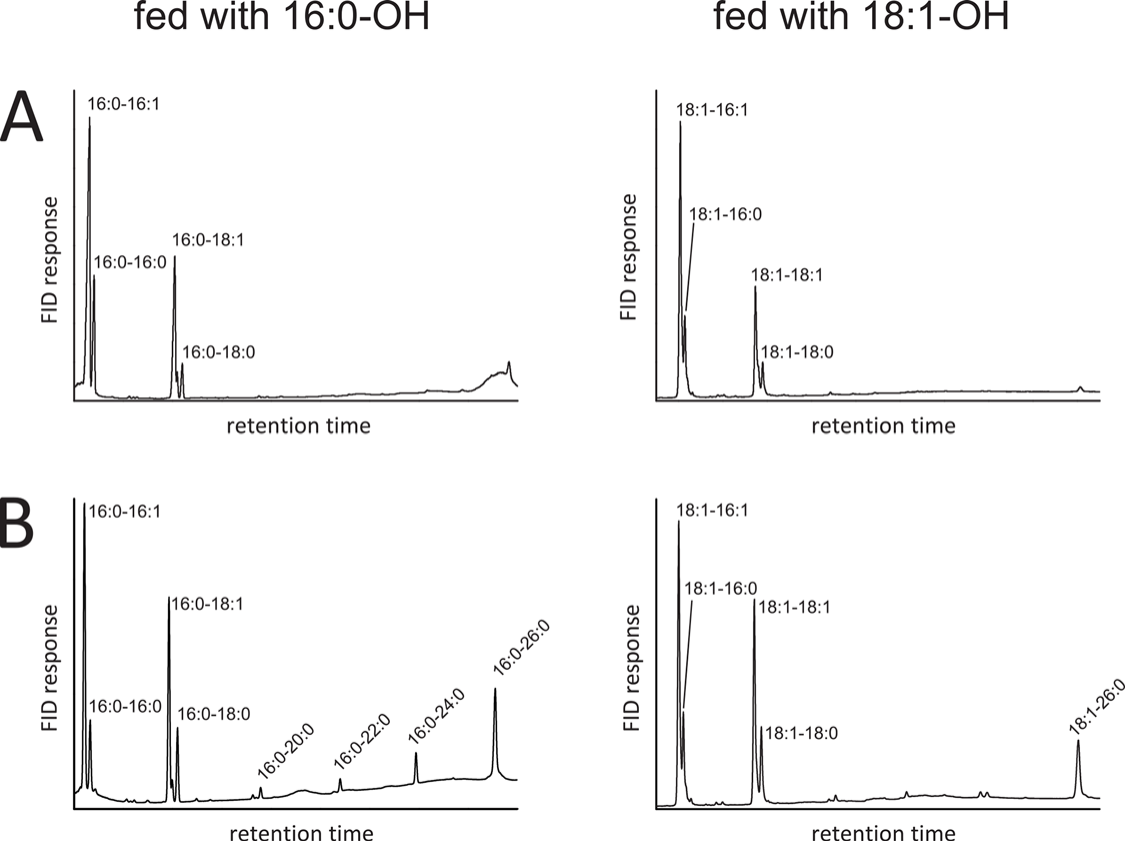
Comparison of WEs derived from yeast cultures expressing AWAT2 or V2. A. GC analysis of WEs derived from a culture expressing mouse AWAT2 either fed with 16:0-OH (left chromatogram) or 18:1-OH (right chromatogram). B. GC analysis of WEs derived from a culture expressing V2 either fed with 16:0-OH (left chromatogram) or 18:1-OH (right chromatogram). In comparison to AWAT2, V2 expressing cultures synthesize four additional WE upon feeding 16:0-OH (left chromatogram) and a single additional WE upon feeding 18:1-OH (right chromatogram). The results shown here for cultures expressing V2 are also representative for cultures expressing V5, mouse AWAT2 N36R as well as AWAT2 A25F N36R (not shown). Data are representative for at least three independent biological replicates.

Interestingly, lipid extracts from cultures expressing V2 and V5, both carrying the TM domains of DGAT2, contained additional lipid species migrating closely above the WE band (Fig 3, arrows). Compared to AWAT2, four additional WE species were observed by GC-FID when respective cultures were supplemented with 16:0-OH (representatively shown for V2 in Fig 4B, left chromatogram). Upon feeding 18:1-OH, only a single additional WE species was observed in comparison to AWAT2 (representatively shown for V2 in Fig 4B, right chromatogram).

**Fig 5:**
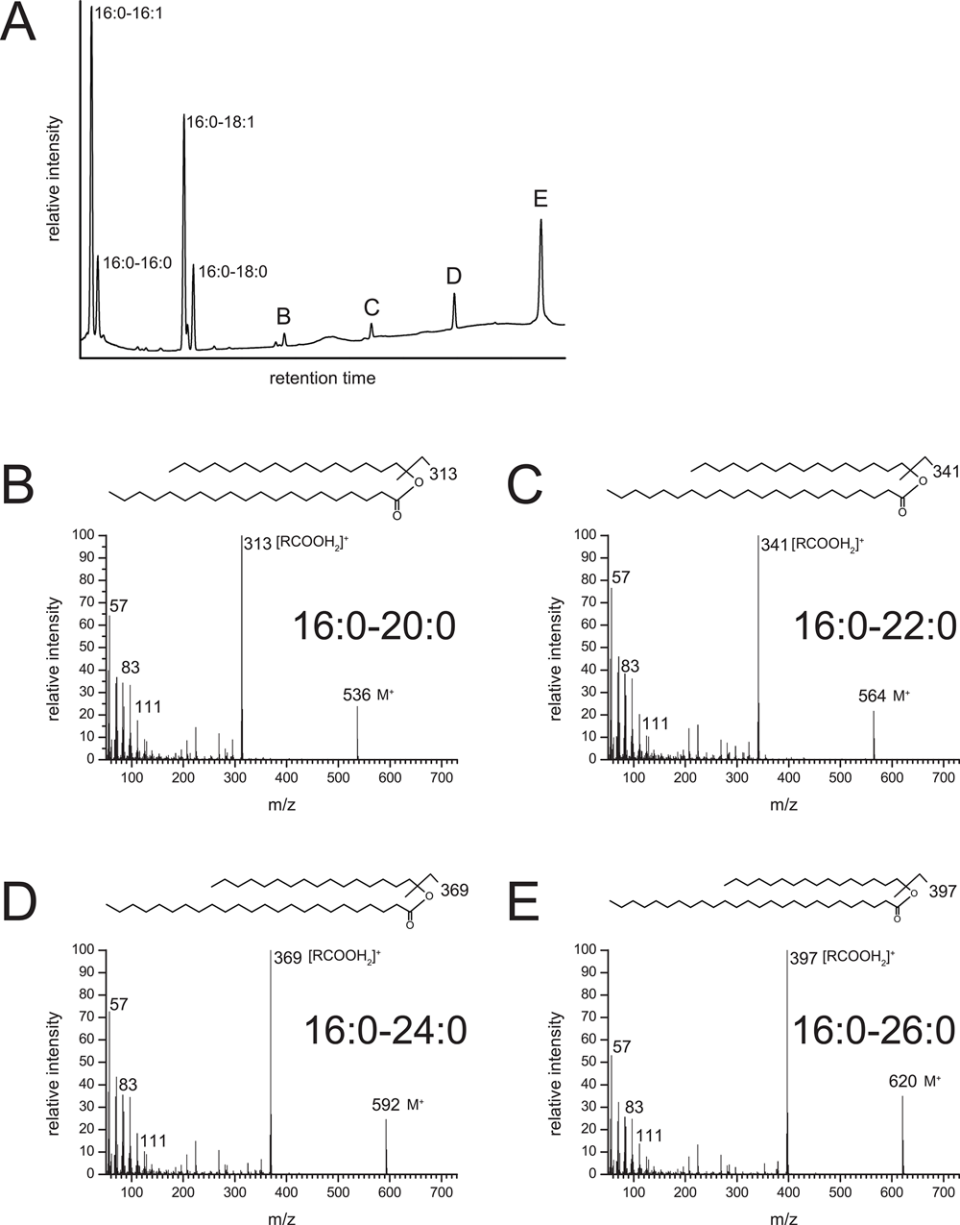
Mass spectra of VLC fatty acyl-harboring WEs produced by mouse cultures expressing V2 fed with 16:0-OH. A. GC analysis of WEs derived from a culture expressing V2 (same chromatogram as in Fig 4B left hand side). B. Mass spectrum of peak (B) identified it as hexadecanoyl-eicosanoate (16:0–20:0), C. mass spectrum of peak (C) identified it as hexadecanoyl-docosanoate (16:0–22:0), mass spectrum of peak (D) identified it as hexadecanoyl-tetracosanoate (16:0–24:0) and mass spectrum of peak (E) identified it as hexadecanoyl-hexacosanoate (16:0–26:0). The results shown here for cultures expressing V2 are also representative for cultures expressing V5, mouse AWAT2 N36R as well as AWAT2 A25F N36R (not shown).

The additional signals in samples from cultures expressing V2 or V5, fed with 16:0-OH, were identified as very long chain WE (VLC WE). In detail, eicosanoyl- (20:0), docosanoyl- (22:0), tetracosanoyl- (24:0) and hexacosanoyl (26:0) chains esterified to 16:0-OH were identified by GC-MS (representatively shown for V2 in Fig 5). The single additional signal in samples from cultures expressing V2 or V5, fed with 18:1-OH, was identified as 18:1–26:0 WE by GC-MS (not shown). In samples derived from AWAT2, only negligible amounts of VLC WE carrying acyl chains longer than 18 carbon atoms (>C18) were found by GC (Fig 4A, Tables 1 and 2).

**Table 1:** Acyl chain composition of WEs synthesized by cultures expressing mouse AWAT2 and mouse AWAT2-derived domain swap variants in comparison with the acyl CoA-pool composition upon feeding of 16:0-OH.

acyl chain	AWAT2	V1	V2	V5	acyl-CoA pool
16:1	0.57 ± 0.03 ^b^	0.50 ± 0.05 ^a^ ^,^ ^b^	0.35 ± 0.04 ^a^	0.41 ± 0.06 ^a^ ^,^ ^b^	0.32 ± 0.06 ^a^
16:0	0.14 ± 0.01 ^b^	0.14 ± 0.02 ^b^	0.08 ± 0.00 ^a^	0.08 ± 0.02 ^a^	0.08 ± 0.01 ^a^
18:1	0.24 ± 0.02 ^b^	0.30 ± 0.03 ^a^	0.27 ± 0.01 ^a^ ^,^ ^b^	0.32 ± 0.05 ^a^	0.32 ± 0.04 ^a^
18:0	0.04 ± 0.01	0.04 ± 0.01	0.08 ± 0.00 ^a^ ^,^ ^b^	0.06 ± 0.01 ^a^ ^,^ ^b^	0.04 ± 0.01
>C18	0.00 ± 0.01 ^b^	0.02 ± 0.01 ^a^ ^,^ ^b^	0.21 ± 0.03 ^a^	0.13 ± 0.03 ^a^ ^,^ ^b^	0.24 ± 0.05 ^a^

^a^ Values are significantly different from respective AWAT2 values as deduced from a student’s t-test (p ≤ 0.05).

^b^ Values are significantly different from respective acyl-CoA pool values as deduced from a student’s t-test (p ≤ 0.05).

Data represent mean and standard deviation of samples derived from at least three independent biological replicates.

**Table 2:** Acyl chain composition of WEs synthesized by cultures expressing mouse AWAT2 and AWAT2-derived domain swap variants in comparison with the acyl CoA-pool composition upon feeding of 18:1-OH.

acyl chain	AWAT2	V1	V2	V5	acyl-CoA pool
16:1	0.54 ± 0.03 ^b^	0.50 ± 0.05 ^b^	0.36 ± 0.04 ^a^	0.39 ± 0.03 ^a^ ^,^ ^b^	0.33 ± 0.06 ^a^
16:0	0.16 ± 0.05 ^b^	0.16 ± 0.03 ^b^	0.13 ± 0.02 ^b^	0.12 ± 0.02 ^b^	0.09 ± 0.01 ^a^
18:1	0.24 ± 0.04	0.28 ± 0.02 ^b^	0.27 ± 0.05	0.27 ± 0.05	0.23 ± 0.03
18:0	0.05 ± 0.01 ^b^	0.05 ± 0.01 ^b^	0.12 ± 0.02 ^a^ ^,^ ^b^	0.09 ± 0.01 ^a^ ^,^ ^b^	0.07 ± 0.01 ^a^
>C18	0.01 ± 0.01 ^b^	0.01 ± 0.01 ^b^	0.13 ± 0.01 ^b^	0.12 ± 0.03 ^a^ ^,^ ^b^	0.28 ± 0.07 ^a^

^a^ Values are significantly different from respective AWAT2 values as deduced from a student’s t-test (p ≤ 0.05).

^b^ Values are significantly different from respective acyl-CoA pool values as deduced from a student’s t-test (p ≤ 0.05).

Data represent mean and standard deviation of samples derived from at least three independent biological replicates.

**Fig 6:**
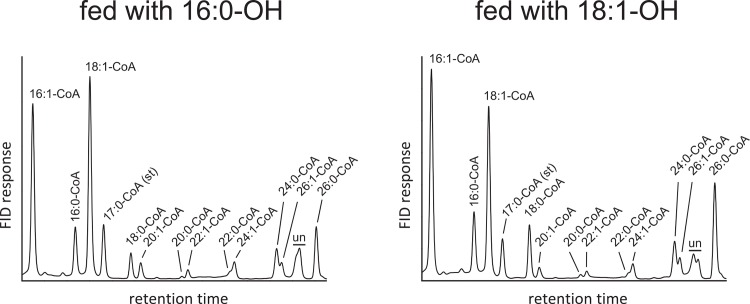
HPLC analysis of acyl-CoA species derived from a culture expressing pYES2/NT (empty vector control) either fed with 16:0-OH (left chromatogram) or 18:1-OH (right chromatogram). un = unidentified). Data are representative for at least three independent biological replicates.

Next, we wanted to relate the formation of VLC WE to the availability of endogenous acyl-CoAs serving as substrates for AWAT2, V2 and V5. In order to evaluate the presence of VLC acyl-CoAs in *S*. *cerevisiae* H1246, the intrinsic acyl-CoA pool of cultures expressing pYES2/NT empty vector control supplemented either with 16:0-OH or 18:1-OH was analyzed by HPLC. Respective measurements indicated the abundance of fully saturated and monounsaturated species of VLC acyl-CoA molecules from 20 to 26 carbon atoms in length (Fig 6). VLC acyl-CoAs longer than C18 comprised for approximately 24% of all measured acyl-CoA species upon feeding of 16:0-OH, while approximately 28% of VLC acyl-CoA were measured in cultures fed with 18:1-OH (Tables 1 and 2). Taken together, VLC acyl-CoAs with a chain length longer than 18 carbon atoms (>C18) accounted for approximately one fourth of all measured species in the acyl-CoA pool of *S*. *cerevisiae* H1246 (Tables 1 and 2). At the same time, WE from cultures expressing AWAT2 did not contain notable amounts of this kind of acyl chains (>C18, Tables 1 and 2). These results may thus suggest that AWAT2 is able to efficiently discriminate VLC acyl-CoA species from WE synthesis and that the different VLC WE-product pattern of V2 and V5 upon feeding either with 16:0 of 18:1 cannot be explained with different substrate pools under these conditions. Moreover, the published preferences of AWAT2 for 16:0-CoA and 16:1-CoA were confirmed, since their amount was almost twice as high in the WE fraction in comparison to the acyl-CoA pool [8,12] that served as substrate for AWAT2 (Tables 1 and 2).

In case of cultures expressing V2, fed with 16:0-OH, VLC fatty acyl-harboring WE species accumulated to about 21% of all WE species (Table 1), whereas in case of feeding 18:1-OH the VLC fatty acyl-harboring WE accumulated to about 13% (Table 2). Hence, V2 and V5 were not able to discriminate VLC acyl-CoA species. In addition, V2 and V5 showed pronounced differences in their overall acyl chain incorporation pattern in comparison to AWAT2, independent of the fed alcohol (Tables 1 and 2). The acyl chain distribution in WEs of cultures expressing either V2 or V5 was rather similar to the acyl chain distribution in the acyl-CoA pool of *S*. *cerevisiae* H1246 expressing pYES2/NT. Only the levels of 18:0 acyl chains in WEs of cultures expressing either V2 or V5 were significantly higher than the respective levels of the acyl-CoA pool (Tables 1 and 2). The acyl chain distribution in WEs from cultures expressing V1 did not show these alterations. It was similar to WEs from cultures expressing AWAT2 and did not contain substantial amounts of VLC WEs (Tables 1 and 2).

### The TM domains of AWAT2 and DGAT2 are highly conserved in vertebrates

**Fig 7:**
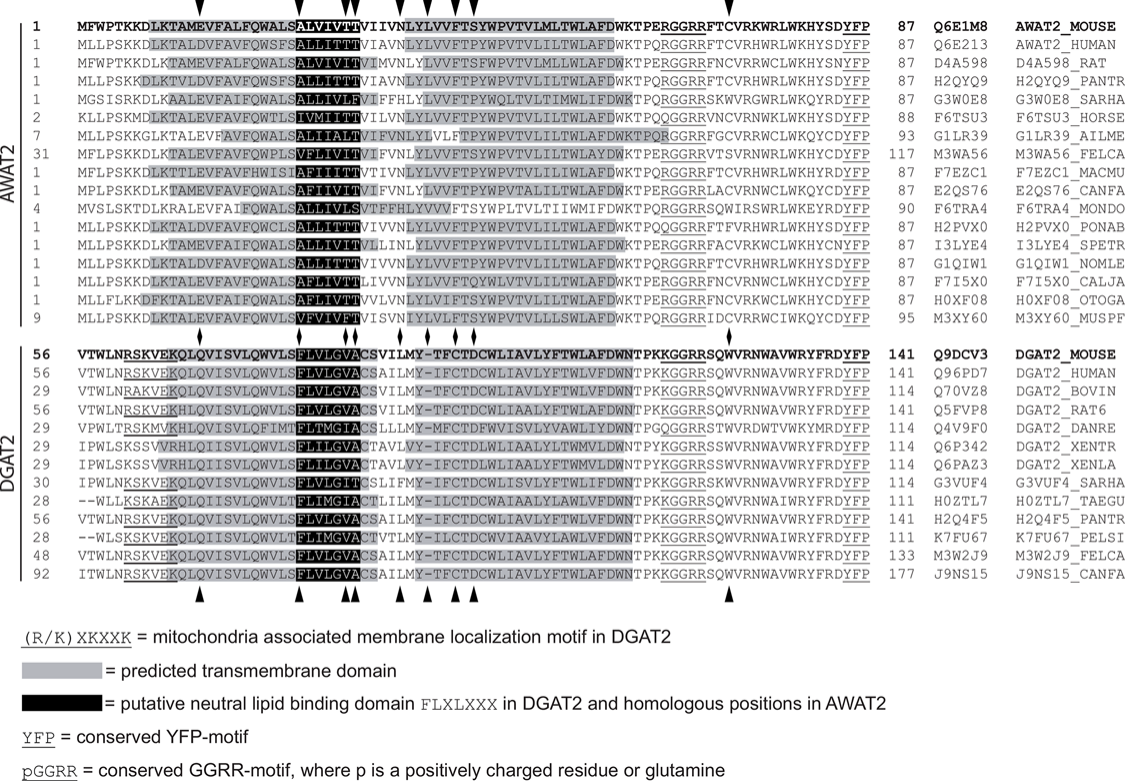
Alignment of the N-terminal part of vertebrate-derived AWAT2 and DGAT2 sequences. Vertebrate-derived sequences were obtained from the UniProt database [37] and aligned using the Clustal Omega tool [38]. The membrane topology was analyzed by using the SOSUI tool [17]. Predicted TM domains are highlighted in gray, whereas parts of the sequence corresponding to the putative neutral lipid binding domain “FLXLXXX” in mouse DGAT2 are highlighted in black. The conserved motifs pGGRR and YFP are underlined. Mouse DGAT2 and AWAT2, which were used in this study, are printed in bold. In case of mouse DGAT2, the indicated TM structure represents the actual topology determined by Stone et al. 2006 [19]. Besides an abbreviation for each enzyme, also the respective UniProt-identifier is listed. A set of 10 conserved amino acid positions were identified, that were different between DGAT2 and AWAT2 sequences. 9 of these positions are in the shown region and marked by arrow heads.

Since the predicted TM domains of AWAT2 had an impact on the substrate specificity of the enzyme, this domain was furthermore analyzed in the available vertebrate-derived AWAT2 and DGAT2 proteins. TM-predictions indicated that the two TM domains were highly conserved among both, AWAT2 and DGAT2, enzymes (Fig 7). Besides that, all sequences harbor a conserved pGGRR motif at the C-terminal side of the second TM domain. Here, p stands for a positively charged residue or a glutamine and the motif thereby represents a cluster of positive charges in the sequence (Fig 7). However, a function of this motif has not been identified yet. Secondly, a YFP motif was detected to be conserved among both AWAT2 and DGAT2 sequences (Fig 7). An exchange of all three residues into alanines was shown to cause an almost complete loss of enzyme activity in DGAT2 of *S*. *cerevisiae* [29]. Two more motifs were only found in DGAT2 enzymes: the putative neutral lipid binding site “FLXLXXX” at the C-terminal end of the first TM domain [25,26] and directly N-terminal to the first TM domain a RXKXXK motif. The latter was found to be responsible for localizing mouse DGAT2 to mitochondria associated membranes [30].

### Changes in substrate specificity of AWAT2 variants can be narrowed down to a single amino acid residue

In order to narrow down the position which may be responsible for the altered substrate specificity of the domain swap variants V2 and V5, a set of 10 conserved amino acids was identified that were conserved in either all DGAT2 or AWAT2 sequences analyzed, but differed between the DGAT2 and AWAT2. For all 10 positions the corresponding exchanges were generated and the products of the resulting mutants were analyzed (Fig 7, 9 of the mutated positions are in the shown region and are marked by arrow heads). At first, the putative neutral lipid binding domain of DGAT2 enzymes was analyzed. In mouse DGAT2, mutating phenylalanine at position 80 into an alanine led to a severely decreased enzyme activity [19]. Interestingly, the corresponding position in AWAT2 is an alanine (Fig 7, second position from the left). Accordingly, it was mutated into a phenylalanine. Analysis of lipid extracts via GC-MS and GC-FID revealed that the AWAT2 A25F variant was still active and was capable of synthesizing small amounts of VLC fatty acyl-harboring WE species. However, these amounts were only little (Tables 3 and 4). A second difference in the putative neutral lipid-binding site between DGAT2 and AWAT2 enzymes is a two threonine motif in approximately half of the AWAT2 sequences (Fig 7, third and fourth position from the left). In DGAT2 sequences, the respective residues were almost exclusively of non-polar nature. However, WEs derived from the resulting AWAT2 T30A T31A mutant were only slightly different from AWAT2 wild type in that they contained little more WEs carrying 16:1 acyl chains, as deduced from a student’s t-test (p<0.05) (S2 Fig).

**Table 3:** Acyl chain composition of WEs synthesized by cultures expressing mouse AWAT2 and AWAT2-derived single amino acid exchange variants in comparison with the acyl CoA-pool composition upon feeding of 16:0-OH.

acyl chain	AWAT2	A25F	A25F N36R	N36R	acyl-CoA pool
16:1	0.57 ± 0.03 ^b^	0.58 ± 0.01 ^b^	0.28 ± 0.01 ^a^ ^,^ ^b^	0.27 ± 0.01 ^a^ ^,^ ^b^	0.32 ± 0.06 ^a^
16:0	0.14 ± 0.01 ^b^	0.09 ± 0.00 ^a^ ^,^ ^b^	0.09 ± 0.00 ^a^ ^,^ ^b^	0.09 ± 0.01 ^a^ ^,^ ^b^	0.08 ± 0.01 ^a^
18:1	0.24 ± 0.02 ^b^	0.27 ± 0.01 ^a^ ^,^ ^b^	0.25 ± 0.01 ^b^	0.22 ± 0.03 ^b^	0.32 ± 0.04 ^a^
18:0	0.04 ± 0.01	0.04 ± 0.00	0.11 ± 0.00 ^a^ ^,^ ^b^	0.12 ± 0.01 ^a^ ^,^ ^b^	0.04 ± 0.01
>C18	0.00 ± 0.01 ^b^	0.02 ± 0.01 ^a^ ^,^ ^b^	0.27 ± 0.00 ^a^	0.30 ± 0.02 ^a^ ^,^ ^b^	0.24 ± 0.05 ^a^

^a^ Values are significantly different from respective AWAT2 values as deduced from a student’s t-test (p ≤ 0.05).

^b^ Values are significantly different from respective acyl-CoA pool values as deduced from a student’s t-test (p ≤ 0.05).

Data represent mean and standard deviation of samples derived from at least three independent biological replicates.

**Table 4:** Acyl chain composition of WEs synthesized by cultures expressing mouse AWAT2 and AWAT2-derived single amino acid exchange variants in comparison with the acyl CoA-pool composition upon feeding of 18:1-OH.

acyl chain	AWAT2	A25F	A25F N36R	N36R	acyl-CoA pool
16:1	0.54 ± 0.03 ^b^	0.48 ± 0.02 ^a^ ^,^ ^b^	0.38 ± 0.02 ^a^	0.32 ± 0.05 ^a^	0.33 ± 0.06 ^a^
16:0	0.16 ± 0.05 ^b^	0.15 ± 0.02 ^b^	0.11 ± 0.01 ^a^ ^b^	0.14 ± 0.02 ^b^	0.09 ± 0.01 ^a^
18:1	0.24 ± 0.04	0.27 ± 0.03 ^b^	0.28 ± 0.02 ^b^	0.24 ± 0.05	0.23 ± 0.03
18:0	0.05 ± 0.01 ^b^	0.07 ± 0.01	0.12 ± 0.01 ^a^ ^,^ ^b^	0.12 ± 0.02 ^a^ ^,^ ^b^	0.07 ± 0.01 ^a^
>C18	0.01 ± 0.01 ^b^	0.03 ± 0.02 ^a^ ^,^ ^b^	0.12 ± 0.04 ^a^ ^,^ ^b^	0.14 ± 0.06 ^a^ ^b^	0.28 ± 0.07 ^a^

^a^ Values are significantly different from respective AWAT2 values as deduced from a student’s t-test (p ≤ 0.05).

^b^ Values are significantly different from respective acyl-CoA pool values as deduced from a student’s t-test (p ≤ 0.05).

Data represent mean and standard deviation of samples derived from at least three independent biological replicates.

**Fig 8:**
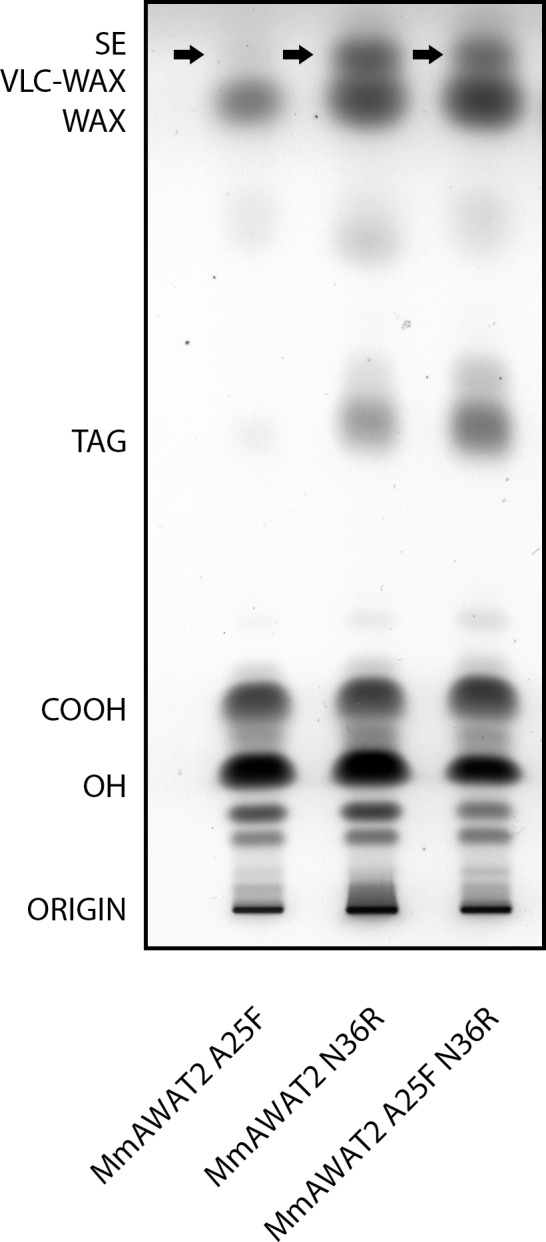
Separation of neutral lipids derived from yeast cultures expressing different mouse AWAT2 variants. All variants were expressed in *S*. *cerevisiae* H1246 and supplemented with 18:1-OH. Similar results were obtained when cultures were supplemented with 16:0-OH (not shown). VLC fatty acyl-containing WEs are indicated by arrows. COOH = free fatty acids, OH = fatty alcohols, TAG = triacylglycerols, WE = wax esters, VLC WE = very long chain fatty acyl-containing wax esters, SE = sterol esters. Data are representative for at least three independent biological replicates.

Apart from the residues homologous to the putative neutral lipid binding site in DGAT2, a conserved polar residue in AWAT2 enzymes is located at the connecting loop of the two TM domains, whereas in vertebrate DGAT2 enzymes, this position is occupied by a non-polar residue throughout (Fig 7, fifth position from the left)). The AWAT2 N36L variant did not show any differences in comparison to AWAT2 upon feeding of 18:1-OH (S2 Fig). In order to evaluate the effect of a residue having an even more polar character than asparagine, the AWAT2 N36R variant, carrying a positive charge, was tested. Cultures expressing the AWAT2 N36R variant were capable of synthesizing VLC fatty acyl-harboring WEs in comparable amounts to the V2 and V5 variants (Fig 8, Tables 3 and 4).

Apart from carrying a positive charge, the arginine in the AWAT2 N36R variant is also rather bulky in comparison to the asparagine residue in AWAT2. To elucidate if the observed effects of the N36R exchange are linked to either the size or the charge of the residue at this position, the AWAT2 N36W and the AWAT2 N36K variants were constructed. The tryptophan in AWAT2 N36W is a non-polar residue and was intended to evaluate the effect of a bulky residue at position 36 of AWAT2. In contrast, lysine is not as big but carries a positive charge like arginine. However, none of the two variants resembled the N36R phenotype (S2 Fig).

As AWAT2 A25F and AWAT2 N36R both showed the ability to synthesize VLC fatty acyl-harboring WEs, the corresponding double variant AWAT2 A25F N36R was tested for additive effects. However, the WE profile of AWAT2 A25F N36R was similar to that of AWAT2 N36R (Fig 8, Tables 3 and 4). Additional variants according to highly conserved differences between AWAT2 and DGAT2 sequences were generated, but none of these exchanges resembled the properties of domain swap variants V2 and V5 or AWAT2 N36R upon feeding with 18:1-OH (S2 Fig). In detail, these additional variants were E14Q, L39-, F42C, S44D, C72W and C106Y (Fig 7).

## Discussion

The aim of this study was to identify substrate specificity determinants in the mouse AWAT2 through comparative studies with the highly similar mouse DGAT2 by expressing the respective enzymes in intact yeast cells that were devoid of synthesizing neutral lipids and were supplemented with fatty alcohol. Both enzymes are able to catalyze the formation of WEs (Fig 3). Using domain swap experiments between AWAT2 and DGAT2, two predicted, neighboring TM domains at the N-terminus of both enzymes were identified to be involved in determination of the substrate specificity of AWAT2. By mutating ten amino acid positions that were conserved in either of the two enzyme families, but were different between AWAT2 and DGAT2 enzymes, an exchange of asparagine 36 to arginine altered chain length specificity for fatty acyl substrates of mouse AWAT2 (Fig 8, Tables 3 and 4). Interestingly, this amino acid is most likely localized in a short luminal stretch connecting the two predicted TM domains (Figs 2A, 2B and 7).

It is tempting to assume that the observed effects may be caused by a loss of substrate specificity in case of the V2, V5 and AWAT2 N36R variants. This lost substrate specificity would be in accordance with the incorporation of VLC acyl-CoAs in WEs, meaning that V2, V5 and AWAT2 N36R may not be able to exclude VLC acyl-CoAs from their active site any more. In addition, this idea may be further supported by the observation that C16 and C18 acyl chains were incorporated in amounts, which were similar to the respective values in the acyl-CoA substrate pool in these variants. However, there are points, which may argue against a loss of substrate specificity. First, the levels of 18:0 acyl chains in WEs derived from cultures expressing either V2, V5 or AWAT2 N36R were approximately twice as high as the levels of 18:0 acyl chains in the acyl-CoA substrate pool (Tables 1–4). Second, cultures, which were fed with 18:1-OH only incorporated 26:0-CoA, whereas 20:0–24:0-CoA were still discriminated (Fig 4). Third, monounsaturated VLC acyl chains were not incorporated in WEs by any of the variants (Figs 4 and 6). Together, these observations suggest that the variants might have at least severely decreased substrate specificities. This effect can be best explained with a disturbed substrate binding mediated by the predicted TM domain.

The observation that V2, V5 and AWAT2 N36R incorporated 20:0–24:0 acyl chains into WEs only upon feeding of 16:0-OH may be explained with differences in conformational changes of the enzyme upon binding of different alcohols. It was already proposed that the reaction mechanism of bacterial WS requires binding of the fatty alcohol moiety as the first substrate in order to protonate the catalytic histidine residue of the active site [9,31]. Under the premise that the mechanisms of bacterial WS and AWAT2 enzymes are comparable, the binding of a fatty alcohol might induce conformational changes in the enzyme which then determine the specificity for the secondly bound acyl-CoA moiety. The observation that mutations in the TM domains of DGAT2 can result in severely decreased enzyme activities [19], while a complete removal of the TM domains results in an active enzyme [32] may further support this model. Mutations in the TM domains as a possible substrate acquiring structure could block the access for substrate to the active center. A complete removal of this binding pocket would in contrast not prevent a general enzymatic activity, yielding to the observed results.

Alternatively, V2, V5 and AWAT2 N36R might interact with different enzymatic partners than the wild type version of AWAT2 does. As a consequence, they might be misallocated in micro domains which harbor high amounts of VLC acyl-CoAs. Direct interaction of different enzymes involved in lipid metabolism via a defined stretch of amino acids in a predicted TM domain was shown for DGAT1 enzymes [33]. Also, it is known that enzymes involved in TAG synthesis can localize to specific micro domains in the ER [34]. Moreover, it was supposed that TAG synthesizing enzymes might form physical complexes [35,36], supporting the general possibility of a differential localization of mutated versions of a protein in micro domains of the ER. However, single amino acid exchanges on the one side and replacement of a complete domain on the other side would have to result in the same changes in enzymatic interaction partners in this case. Alternatively, VLC fatty acyl-harboring WE producing variants might have lost the ability to interact with certain enzymatic partners AWAT2 interacts with. As a consequence, they would be released from a micro domain containing no VLC acyl-CoAs in regions of the ER in which VLC acyl-CoAs are present. However, the observation that the mouse DGAT2 wild type enzyme harbors the same TM domains as the V2 and V5 variants, but does not use VLC acyl-CoAs as substrates renders this third hypothesis unlikely as well. Taken together the data presented here strongly suggest that a predicted TM domain of mouse AWAT2 determines the enzyme’s substrate specificity or is even involved in substrate binding. However, we would like to point out that all conclusions presented here derive from data obtained from expressing the enzymes in a yeast mutant that was supplemented with fatty alcohol. *In vitro* enzymatic assays to test the enzymatic properties of the various chimeric proteins in a different assay system were considered. To our experience, such assays are only helpful if substrates of similar chain lengths are compared. However, the acyl-CoAs that need to be tested here differ in up to 10 carbon atoms and therefore have strong differences in solubility, which may distort the results of these assays.

To our knowledge, this work is the first study providing insights into structure-function relationships in AWAT-type WS to date. As discussed above and shown in Fig 7, the N-terminal predicted TM-domains in mouse AWAT2 and DGAT2 are highly conserved among other AWAT2 and DGAT2 enzymes. Thus, it may well be that the findings of the present study also apply for respective enzymes from other organisms. The study might thus contribute to the understanding of substrate specificity determining structures and mechanisms of AWAT-type WS in order to be able to design enzymes with tailored substrate specificities for biotechnological uses in the future.

## Supporting Information

S1 Fig Oligonucleotides used for cloning and mutagenesis of mouse WAT2, mouse DGAT2, domain swap variants and single amino acid exchange variants of mouse AWAT2. (DOCX)

S2 Fig Acyl chain composition of WEs synthesized by cultures expressing mouse AWAT2 and mouse AWAT2-derived variants in comparison with the acyl CoA-pool composition upon feeding of 18:1-OH. (DOCX)

## Abbreviations

AWAT: acyl-CoA:wax alcohol acyltransferase
DAG: diacylglycerol
DGAT: acyl-CoA:diacylglycerol O-acyltransferase
TAG: triacylglycerol
TLC: thin layer chromatography
TM: transmembrane
VLC: very long chain
WE: wax ester
WS: wax synthase
