# Supplementary material for: Two Predicted Transmembrane Domains Exclude Very Long Chain Fatty acyl-CoAs from the Active Site of Mouse Wax Synthase
Source: PLoS One. 2015 Dec 29;10(12):e0145797. doi: 10.1371/journal.pone.0145797 (PMC4694924; doi:10.1371/journal.pone.0145797)
Supplement: S1 Fig — (DOCX) [file pone.0145797.s001.docx]

**S1 Fig. Oligonucleotides used for cloning and mutagenesis of mouse WAT2, mouse DGAT2, domain swap variants and single amino acid exchange variants of mouse AWAT2.**

| **CONSTRUCT** | **FORWARD** | **REVERSE** |
| --- | --- | --- |
| AWAT2 | 5‑ACGGGATCCATGTTCTGGCCTACTAAG-3 | 5‑ACGCTCGAGTTAGACGATAACCAGCTC-3 |
| DGAT2 | 5‑ACGGGATCCATGAAGACCCTCATCGC-3 | 5‑ACGCTCGAGTCAGTTCACCTCCAGCA-3 |
| V1 (DGAT2-part) | 5‑ACGGGATCCATGAAGACCCTCATCGC-3 | 5‑AAAGACCTCCATGGCCTGCAGCTGTTTTTCCAC-3 |
| V1 (AWAT2-part) | 5‑GAAAAACAGCTGCAGGCAATGGAGGTTTTCGCT-3 | 5‑ACGCTCGAGTTAGACGATAACCAGCTC-3 |
| V2 (DGAT2-part) | 5‑ACGGGATCCATGTTCTGGCCTACTAAGAAGGACCTTAAGACTCAGCTGCAGGTCATCTCA-3 | 5‑TCTACCTCCTCTTTCGGGCGTGTTCCAGTCAAA-3 |
| V2 (AWAT2-part) | 5‑GACTGGAACACGCCCGAAAGAGGAGGTAGAAGG-3 | 5‑ACGCTCGAGTTAGACGATAACCAGCTC-3 |
| V3 (AWAT2-part) | 5‑ACGGGATCCATGTTCTGGCCTACTAAG-3 | 5‑CCTTCTACCTCCTTTTTTAGGTGTTTTCCAATCGAATGC-3 |
| V3 (DGAT2-part) | 5‑GGAAAACACCTAAAAAAGGAGGTAGAAGGTCGCAGTGGG-3 | 5‑ACGCTCGAGTCAGTTCACCTCCAGCA-3 |
| V4 (AWAT2-part 1) | 5‑ACGGGATCCATGTTCTGGCCTACTAAG-3 | 5‑CGCACCCACTGCGACCTTCTACCTCCTCTTTCA-3 |
| V4 (DGAT2-part) | 5‑GAGGAGGTAGAAGGTCGCAGTGGGTGCGAAACT-3 | 5‑GATACCAGGGAAGGTCTTGCTGACTTCAGTAGC-3 |
| V4 (AWAT2-part 2) | 5‑TACTGAAGTCAGCAAGACCTTCCCTGGTATCACAC-3 | 5‑ACGCTCGAGTTAGACGATAACCAGCTC-3 |
| V5 (DGAT2-part) | 5‑ACGGGATCCATGAAGACCCTCATCGC-3 | 5‑CTCACACATGTGAATCTCCTGCCACCTTTCTTG-3 |
| V5 (AWAT2-part) | 5‑GAAAGGTGGCAGGAGATTCACATGTGTGAGAAAG-3 | 5‑ACGCTCGAGTTAGACGATAACCAGCTC-3 |
| V6 (AWAT2-part) | 5‑ACGGGATCCATGTTCTGGCCTACTAAG-3 | 5‑GGGCAGATGCCTCCTGACATGACGTAATCTCTC-3 |
| V6(DGAT2-part) | 5‑GATTACGTCATGTCAGGAGGCATCTGCCCTGTC-3 | 5‑ACGCTCGAGTCAGTTCACCTCCAGCA-3 |
| V7 (AWAT2-part) | 5‑ACGGGATCCATGTTCTGGCCTACTAAG-3 | 5‑AAGTGGGAACCAGAGAAACCCCATGCTTTAGTGC-3 |
| V7(DGAT2-part) | 5‑GCATGGGGTTTCTCTGGTTCCCACTTATTCCTTT-3 | 5‑ACGCTCGAGTCAGTTCACCTCCAGCA-3 |
| AWAT2 E14Q | 5‑GACTGCAATGCAGGTTTTCGCTCTTTTCCAATGGG-3 | 5‑GAGCGAAAACCTGCATTGCAGTCTTAAGGTCC-3 |
| AWAT2 A25F | 5‑TGGGCTTTGTCTTTCCTCGTTATCGTTACTACCGTC-3 | 5‑AACGATAACGAGGAAAGACAAAGCCCATTGGAAAAGAGCG-3 |
| AWAT2 T30A T31A | 5‑CGTTATCGTTGCTGCCGTCATCATCGTGAACCTGTACC-3 | 5‑CGATGATGACGGCAGCAACGATAACGAGTGCAGAC-3 |
| AWAT2 N36K | 5‑CCGTCATCATCGTGAAACTGTACCTTGTTGTGTTCAC-3 | 5‑CACAACAAGGTACAGTTTCACGATGATGACGGTAGTAACG-3 |
| AWAT2 N36L | 5‑CCGTCATCATCGTGCTCCTGTACCTTGTTGTGTTCAC-3 | 5‑CAAGGTACAGGAGCACGATGATGACGGTAGTAACG-3 |
| AWAT2 N36R | 5‑CGTCATCATCGTGCGCCTGTACCTTGTTGTGTTCAC-3 | 5‑CAAGGTACAGGCGCACGATGATGACGGTAGTAACG-3 |
| AWAT2 N36W | 5‑CCGTCATCATCGTGUGGCTGTACCTTGTTGTGTTCAC-3 | 5‑CACAACAAGGTACAGCCACACGATGATGACGGTAGTAACG-3 |
| AWAT2 L39- | 5‑CGTGAACCTGTACGTTGTGTTCACTTCATACTGGCC-3 | 5‑GTGAACACAACGTACAGGTTCACGATGATGACGG-3 |
| AWAT2 F42C | 5‑CCTTGTTGTGTGCACTTCATACTGGCCAGTTACC-3 | 5‑CAGTATGAAGTGCACACAACAAGGTACAGGTTCACG-3 |
| AWAT2 S44D | 5‑GTTGTGTTCACTGACTACTGGCCAGTTACCGTTTTGATGC-3 | 5‑GGTAACTGGCCAGTAGTCAGTGAACACAACAAGGTACAGG-3 |
| AWAT2 C72W | 5‑GGTAGAAGGTTCACATGTGGGAGAAAGTGGCGTCTTTGG-3 | 5‑CACTTTCTCACCCATGTGAACCTTCTACCTCCTCTTTCAGG-3 |
| AWAT2 C106Y | 5‑TACATACTTGTCTATCACCCACATGGTCTTATGGCAC-3 | 5‑GACCATGTGGGTGATAGACAAGTATGTAGTTTCTATCAGG-3 |
