# Supplementary material for: Two Predicted Transmembrane Domains Exclude Very Long Chain Fatty acyl-CoAs from the Active Site of Mouse Wax Synthase
Source: PLoS One. 2015 Dec 29;10(12):e0145797. doi: 10.1371/journal.pone.0145797 (PMC4694924; doi:10.1371/journal.pone.0145797)
Supplement: S2 Fig — (DOCX) [file pone.0145797.s002.docx]

S2 Fig. Acyl chain composition of WEs synthesized by cultures expressing mouse AWAT2 and mouse AWAT2-derived variants in comparison with the acyl CoA-pool composition upon feeding of 18:1-OH.

| acyl chain | MmAWAT2 | E14Q | T30A T31A | N36K | N36L | N36W | L39- | F42C | S44D | C72W | C106Y | acyl-CoA pool |
| --- | --- | --- | --- | --- | --- | --- | --- | --- | --- | --- | --- | --- |
| 16:1 | 0.54 ± 0.03 ^b^ | 0.45 ± 0.04 ^a^ | 0.58 ± 0.02 ^a, b^ | 0.45 ± 0.05 ^a, b^ | 0.56 ± 0.02 ^b^ | 0.50 ± 0.01 ^a, b^ | 0.57 ± 0.02 ^b^ | 0.50 ± 0.04 ^b^ | 0.48 ± 0.01 ^a, b^ | 0.52 ± 0.00 ^b^ | 0.54 ± 0.06 ^b^ | 0.33 ± 0.06 ^a^ |
| 16:0 | 0.16 ± 0.05 ^b^ | 0.21 ± 0.03 ^b^ | 0.17 ± 0.01 ^b^ | 0.16 ± 0.01 ^b^ | 0.18 ± 0.01 ^b^ | 0.17 ± 0.01 ^b^ | 0.17 ± 0.02 ^b^ | 0.18 ± 0.03 ^b^ | 0.19 ± 0.00 ^b^ | 0.19 ± 0.04 ^b^ | 0.17 ± 0.01 ^b^ | 0.09 ± 0.01 ^a^ |
| 18:1 | 0.24 ± 0.04 | 0.27 ± 0.01 | 0.20 ± 0.01 ^b^ | 0.27 ± 0.03 | 0.21 ± 0.02 | 0.23 ± 0.01 | 0.22 ± 0.00 | 0.21 ± 0.02 | 0.27 ± 0.00 ^b^ | 0.22 ± 0.02 | 0.20 ± 0.04 | 0.23 ± 0.03 |
| 18:0 | 0.05 ± 0.01 ^b^ | 0.06 ± 0.01 | 0.05 ± 0.01 ^b^ | 0.10 ± 0.03 ^a^ | 0.05 ± 0.00 ^b^ | 0.08 ± 0.01 ^a^ | 0.05 ± 0.00 ^b^ | 0.07 ± 0.01 | 0.05 ± 0.00 ^b^ | 0.06 ± 0.01 | 0.07 ± 0.02 | 0.07 ± 0.01 ^a^ |
| >C18 | 0.01 ± 0.01 ^b^ | 0.00 ± 0.00 ^b^ | 0.00 ± 0.00 ^b^ | 0.01 ± 0.01 | 0.00 ± 0.00 ^b^ | 0.02 ± 0.01 ^a, b^ | 0.00 ± 0.00 ^b^ | 0.03 ± 0.02 ^b^ | 0.00 ± 0.00 ^b^ | 0.01 ± 0.00 ^b^ | 0.01 ± 0.01 ^b^ | 0.28 ± 0.07 ^a^ |

^a^ Values are significantly different from respective MmAWAT2 values as deduced from a student’s t-test (p ≤ 0.05).

^b^ Values are significantly different from respective acyl-CoA pool values as deduced from a student’s t-test (p ≤ 0.05).

Data represent mean and standard deviation of samples derived from at least three biological replicates.
